# Synaptic plasticity in cocaine-seeking ensembles of the nucleus accumbens core

**DOI:** 10.1038/s41386-026-02425-y

**Published:** 2026-05-02

**Authors:** Levi T. Flom, Skylar L. Hodgins, German Gutierrez Erives, Jordan M. Russelavage, Samuel M. Hyken, Zhaojie Zhang, Christopher E. Vaaga, Ana-Clara Bobadilla

**Affiliations:** 1 Department of Biomedical Sciences, Colorado State University, Fort Collins, CO USA; 2 School of Pharmacy, University of Wyoming, Laramie, WY USA; 3 Department of Neuroscience, Medical University of Southern Carolina, Charleston, SC USA; 4 Department of Zoology and Physiology, University of Wyoming, Laramie, WY USA

**Keywords:** Short-term potentiation, Reward, Classical conditioning

## Abstract

**Abstract:**

Cue-induced seeking engages neuronal ensembles within the nucleus accumbens core (NAcore), with neuronal ensembles defined here as neurons coactivated during specific behavioral experiences that have been implicated in cued-reinstatement. Although transient synaptic plasticity has been widely observed in unidentified ensemble and non-ensemble neuronal populations in the NAcore during reinstatement, its expression within behaviorally relevant ensembles remains unclear. Here, we used c-Fos-TRAP2-based tagging to characterize structural and functional synaptic plasticity within ensembles during cocaine-seeking in mice following cocaine intravenous self-administration, extinction, and cue-induced reinstatement. Structural plasticity was measured via spine confocal imaging, and functional changes were evaluated by AMPA/NMDA ratios using whole-cell electrophysiology across reinstatement time points. Ensemble neurons exhibited increased dendritic spine head diameter during cue-induced reinstatement and were functionally potentiated relative to non-ensemble neurons. Spine classification showed reduced mature spines during reinstatement in both ensemble and non-ensemble cells, suggesting morphological remodeling rather than new spine formation. Non-ensemble neurons showed no change in spine head diameter during reinstatement but did exhibit an increased AMPA/NMDA ratio during cued-reinstatement. Paired-pulse ratio analysis suggested that yoked-cocaine exposure decreased presynaptic vesicle release probability, while operant cocaine exposure had no effect. Ensemble neurons showed an elevated AMPA/NMDA ratio following cocaine exposure, regardless of whether intake was yoked or contingent. Together, these findings suggest that ensemble and non-ensemble neurons undergo distinct forms of synaptic plasticity during cue-induced reinstatement. By distinguishing ensemble-specific structural plasticity from non-ensemble functional plasticity, this study refines the current understanding of mechanisms underlying cue-induced relapse.

**Significance Statement:**

In preclinical models of substance use disorder drug seeking is associated with cue-induced reactivation of neuronal ensembles in the nucleus accumbens core. While transient synaptic plasticity has been extensively described in non-selective neuronal populations pooling recordings of both ensemble and non-ensemble neurons of the nucleus accumbens core, ensemble-specific plasticity remains unclear. Here, we combined c-Fos-TRAP2 tagging, confocal imaging, and slice electrophysiology to show that structural synaptic plasticity is selectively expressed in behaviorally relevant ensembles. By linking ensemble identity with structural and functional plasticity during cue-induced cocaine seeking, these findings refine current models of relapse and identify plasticity within the ensemble as a potential target for therapeutic intervention.

## Introduction

In substance use disorders (SUDs), cue-evoked relapse engages the nucleus accumbens core (NAcore), a key integrator of glutamatergic reward circuitry [1, 2]. In preclinical models of SUD, cue-induced reinstatement reliably engages the NAcore glutamatergic circuitry that drives relapse-like behavior [3]. Neuronal ensembles, defined as sparsely distributed neurons activated by specific behavioral experiences, are a focus of SUD research [4–6], aided by activity-dependent tagging approaches such as TRAP (Targeted Recombination in Active Populations) [7], which enables permanent labeling of ensembles throughout the brain [5, 8–11].

Many studies examine dendritic spine plasticity during reinstatement [12]. Cue-induced reinstatement correlates with transient synaptic plasticity in the NAcore during cocaine, nicotine, and heroin seeking [13–15]. Transient synaptic plasticity involves increased spine head diameter and an AMPA/NMDA ratio that peaks within 15–30 minutes and resolves by 2 hours of cued reinstatement [14, 16]. Because these studies used only male rats and non-selective recordings that mixed ensemble and non-ensemble cells, it remains unclear whether similar adaptations occur in females or in defined ensembles.

Using c-Fos-TRAP2 tagging in male and female mice, we examined structural and functional transient synaptic plasticity in ensemble and non-ensemble NAcore neurons following cocaine self-administration and cue-induced reinstatement. We hypothesized that ensemble and non-ensemble neurons exhibit distinct structural and functional plasticity during cocaine seeking.

## Methods

### Animals

Female (*n* = 21) and male (*n* = 34) Ai14 x c-Fos-TRAP2 transgenic mice were generated by crossing tamoxifen-inducible c-Fos-iCre knock-in males (Fostm2.1(icre/ERT2)Luo/J, JAX #030323, RRID: IMSR_JAX:030323) with Ai14 iCre-reporter females (B6;Cg-Gt(ROSA)26Sortm14(CAG-tdTomato)Hze/J, JAX #007914, RRID: IMSR_JAX:007914). Mice were singly housed on a 12:12 reverse light cycle. Sample size and attrition were based on established methods [5]. All procedures followed NIH guidelines and were approved by the IACUCs at the University of Wyoming and Colorado State University. Final cohorts included *n* = 36 structural (virus + catheter), *n* = 19 functional (catheter only), all contributing to behavioral data (*n* = 55; cocaine *n* = 37, yoked-saline *n* = 12, yoked-cocaine *n* = 6). Final sample sizes reflect exclusions detailed in Supplemental Materials.

### Surgery

8- to 22-week-old male and female mice underwent stereotaxic microinjections and jugular catheterization under isoflurane anesthesia. Catheters were secured to the jugular vein with infusion ports fixed to the skull. For structural cohorts, 300 nl of diluted pAAV-hSyn-eGFP (2.15 × 10^12^ GC/ml) was bilaterally injected into the NAcore (AP + 1.5–1.6 mm, ML ± 1.0 mm, DV − 4.4 mm). Functional cohorts received catheterization only. Mice recovered for 4 days with heparin flushes, carprofen, and topical antibiotics (see Supplementary Methods).

### Cocaine self-administration, extinction, and reinstatement

**Fig. 1:**
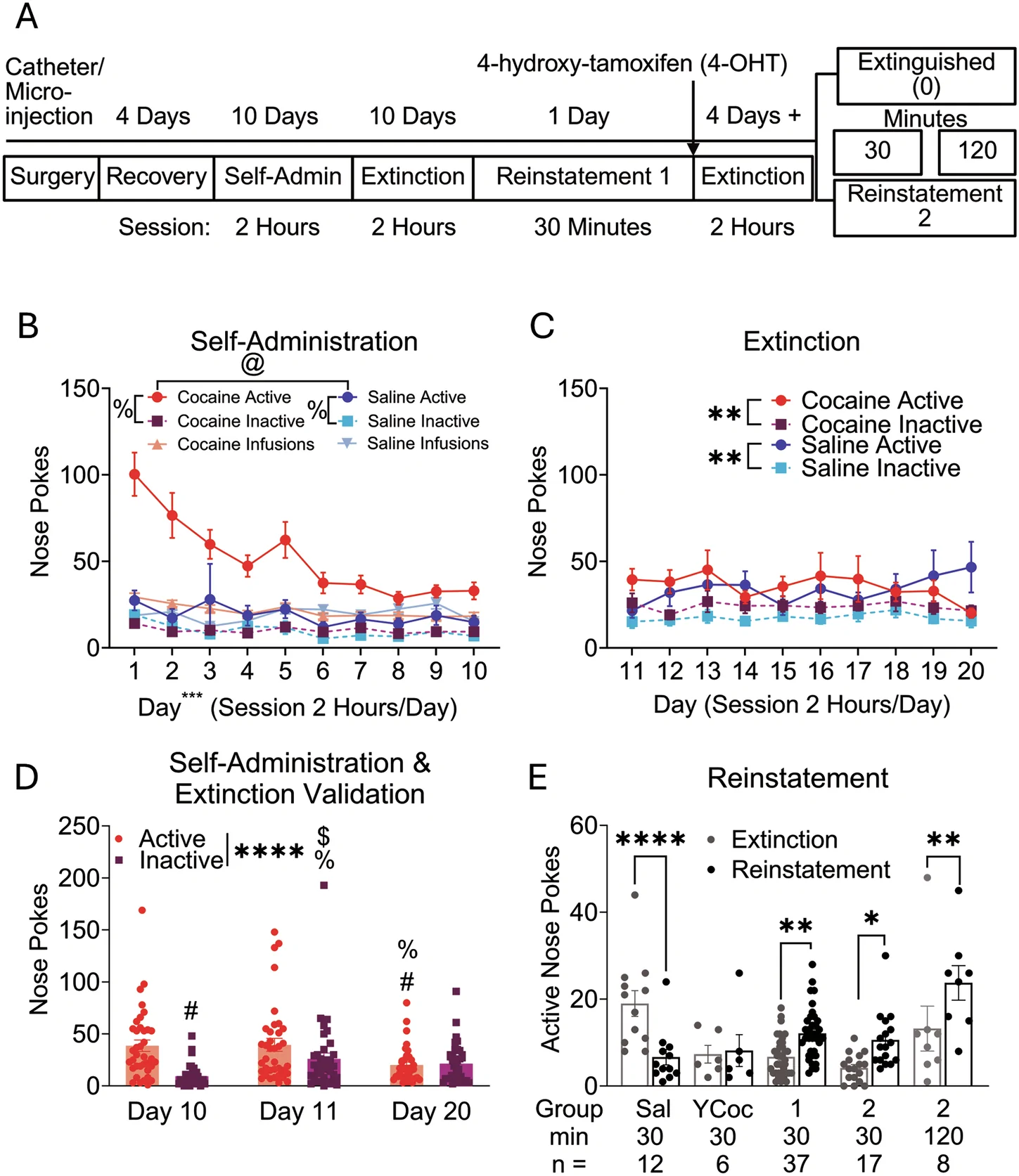
Cocaine self-administration behavior. **A** Experimental timeline. Animals were injected with 4-hydroxy-tamoxifen (4-OHT) following the first reinstatement. **B** Nose-poking behaviors of cocaine and yoked-saline controls. ***p < 0.001 difference across days, %*p* < 0.0001 active vs inactive nose-pokes, @*p* < 0.0001 difference between cocaine and yoked-saline animals. **C** Extinction behavior in mice shows active nose-pokes were different compared to inactive nose-pokes in both groups, **p < 0.01. **D** Validation of behavioral changes shows that animals extinguished behavior. #*p* < 0.01 compared to day 10 active nose-poke, $*p* < 0.05 compared to day 10 inactive, %*p* < 0.01 compared to day 11 active nose-poke. **E** Reinstatement behavior in mice shows no association with yoked controls but shows seeking in cocaine animals. *p < 0.05, **p < 0.01, ****p < 0.0001.

Mice self-administered cocaine (0.5 mg/kg/inf) or were assigned to yoked-saline or yoked-cocaine control groups during 2‑h daily sessions for 10 consecutive days in operant chambers equipped with active/inactive nose-poke ports and a cue light above the active port (Fig. 1A). For the cocaine self-administration group, active nose-pokes resulted in intravenous cocaine infusion paired with illumination of a cue light within the active port for 3 s, followed by a 10-s timeout during which the cue light was off, and additional responses had no programmed consequences. Yoked animals had no control over infusions and received an infusion of saline or cocaine temporally matched to a paired cocaine self-administering mouse. Yoked-saline controls accounted for the infusion procedure and handling [16], whereas yoked-cocaine controls isolated cocaine pharmacology exposure independent of contingency. To facilitate acquisition [17–19], mice were maintained on a restricted diet that provided 80% of normal intake. Mice were considered to have acquired cocaine self-administration if they earned an average of ≥10 cocaine infusions per session across the 10 training days and directed ≥60% of their nose-poke responses to the active port during the final five sessions. These criteria ensured robust intake and stable nose-poke discrimination during the final sessions, even though mice typically show greater behavioral variability than rats [20, 21]. Of 39 cocaine self-administration animals, two animals failing to meet these criteria were excluded from further analysis.

Following self-administration, extinction consisted of daily 2-hour sessions in which nose-pokes had no programmed consequences (no cocaine infusion or cue presentation). Extinction continued for a minimum of 10 days until mice met the extinction criterion, defined as a ≥70% decrease in nose-poke responding relative to baseline for the first 30 min of the last extinction session. This time window was selected to allow comparison with 30-min reinstatement sessions, as goal-directed responding is typically highest at session onset [22].

Cue‑induced reinstatement 1 was conducted the day after the extinction criteria were met. Immediately following reinstatement 1, mice received 4‑hydroxytamoxifen (4‑OHT; 50 mg/kg, i.p.). This dose was chosen based on previous studies [5, 7, 8, 23] to enable activity-dependent TRAPing of neurons during reinstatement. Ensemble neurons were defined as tdTomato‑positive (tdTomato+) neurons labeled during reinstatement 1. Non‑ensemble neurons were defined as GFP‑expressing neurons in structural cohorts or, for electrophysiology, tdTomato‑negative neurons within the same NAcore regions. Extinction training resumed 24 h later for a minimum of four additional sessions to reduce responding before reinstatement 2 and to allow tdTomato expression.

On the test day, mice assigned to the 30-min and 120-min reinstatement 2 groups underwent a cue-induced reinstatement 2 session and were euthanized immediately after session completion. The 0-min extinguished group and yoked controls remained in their home cages and were euthanized without additional chamber exposure, but were otherwise handled identically to the reinstatement 2 group, as in prior transient plasticity studies [14, 24]. Previous TRAP studies reported 45–60% reactivation between reinstatement sessions, based on tdTomato and c-Fos IHC colabeling [5], consistent with ensemble reactivation during memory retrieval [7, 25, 26]. This indicates that TRAPed neurons are preferentially re-engaged during reinstatement 2.

### Immunohistochemistry

Mice were perfused with phosphate-buffered saline (PBS, pH 7.4) followed by 3.7% formaldehyde in PBS. Brains were post-fixed, cryoprotected, and sectioned at 80 µm. Free-floating sections were blocked and incubated with rabbit anti-RFP antibody (1:1000) and chicken anti-GFP antibody (1:10,000), followed by Alexa Fluor secondary antibodies (594, 488). Sections were mounted with Prolong Gold (see Supplementary Methods).

### Imaging and spine analysis

Confocal images of NAcore medium spiny neurons (Bregma +1.7 to +0.62 mm) were acquired using Zeiss LSM 880/980 microscopes (63× oil, voxel size 0.164 µm XY, 0.25 µm Z). Four dendritic segments per neuron-type (30–110 µm, ≥50 µm from soma) were imaged, and images were analyzed in Imaris (v10). Spines were identified using semi-automated filament tracing and classified (stubby, mushroom, thin) with the Imaris XT plugin [27]. Spine density and head diameter metrics were calculated from all detected spines.

### Electrophysiology

Acute NAcore slices (250–300 μm) were prepared from p85–117 male and female mice. Animals were anesthetized with isoflurane (2–3%) and transcardially perfused with cold, oxygenated cutting solution in mM: 83 NaCl, 2.5 KCl, 1 NaH_2_PO_4_, 26.2 NaHCO_3_, 22 dextrose, 72 sucrose, 3.3 MgCl_2_, 0.5 CaCl_2_, (290–310 mosmol, pH 7.3). Slices were recovered for ≥30 min in 37 °C artificial cerebrospinal fluid (in mM: 123 NaCl, 3.5 KCl, 1.25 NaHPO_4_, 26 NaHCO_3_, 1 MgCl_2_, 1.5 CaCl_2_, 10 glucose).

Whole‑cell voltage clamp recordings were obtained from tdTomato‑positive and tdTomato-negative NAcore cells using an Olympus BX51WI microscope with CoolLED (550 nm) illumination. GABA_A transmission was blocked with SR95531 (20 μM). Pipettes (2–5 MΩ) were filled with Cs‑gluconate internal solution (in mM: 120 CsCH_3_SO_3_, 3 NaCl, 2 MgCl_2_, 1 EGTA, 10 HEPES, 4 MgATP, 0.3 Tris‑GTP, 14 Tris‑creatine phosphate, 12 sucrose; 288 mosmol, pH 7.32). Recordings were corrected for a 10 mV junction potential, maintained at 35–37 °C, digitized at 20 kHz, and filtered at 10 kHz (Sutter IPA amplifier, SutterPatch/IgorPro). Before and after data acquisition, access resistance was recorded using a 10-mV hyperpolarizing step. Cells with > 20% change in series resistance during recordings were excluded from analysis.

Cortical afferents were stimulated with a bipolar electrode placed medial/dorsal to the anterior commissure (0.1 ms, 2–32 mA). Excitatory postsynaptic currents were recorded at −70 mV (AMPA) and +40 mV (NMDA), averaged over ≥10 sweeps. AMPA/NMDA ratios were calculated as peak AMPA amplitude (−70 mV) divided by NMDA amplitude (+40 mV, 40 ms post‑stimulus).

### Experimental design and statistics

Behavioral data (self-administration, extinction) were analyzed in Prism v10 using three‑way repeated measure (RM) ANOVA with Geisser‑Greenhouse correction (group [cocaine vs yoked-saline] × nose-poke [active vs inactive] × days). A mixed-effect model with planned comparisons was used to analyze reinstatement. Sex effects were assessed separately with three‑way ANOVA and post‑hoc comparisons of active vs inactive nose-poke.

Structural data were validated by comparing tdTomato vs GFP spine density/diameter with nested t‑tests, followed by nested ANOVA and Tukey’s post‑hoc tests. Segment averages were collapsed to animal means for two‑way ANOVA (population [ensemble vs non-ensemble] × group [yoked-cocaine, yoked-saline, extinguished 0 min, 30 min, and 120 min]). Spine type assays were analyzed similarly. Sex effects on structural data were tested with two‑way ANOVA (sex × population/group).

Functional data treated each cell as independent. AMPA/NMDA ratios were compared between groups initially with one-way ANOVA and ensemble vs non‑ensemble cells exposed to cocaine using an unpaired *t*‑test, then analyzed with two‑way ANOVA (population × group [yoked-cocaine, yoked-saline, extinguished 0 min, and 30 min]) with Tukey’s post‑hoc tests. Paired‑pulse ratio (PPR) was assessed with the same design. Regression analyses examined relationships between reinstatement nose-poke, spine diameter, and AMPA/NMDA ratio.

Adjusted degrees of freedom are reported for Geisser-Greenhouse corrections. Post hoc comparisons were performed using Tukey’s tests unless otherwise specified, and all *p*-values reflect corrected multiple comparisons. As no significant sex effects were observed, data are presented collapsed across sex unless otherwise noted.

## Results

### Cocaine self-administration, extinction, and reinstatement behavior

We assessed cocaine self-administration across training days. There was a main effect of days (*p* = 0.0006, Fig. 1B), group (*p* < 0.0001), nose-poke (*p* < 0.0001), and interaction of nose-poke × group (*p* < 0.0001). Full statistical results provided in ST1. Thus, operant responding changed during self-administration and differed between groups (yoked-saline vs. cocaine). The gradual reduction in active nose-poke responding across sessions likely reflected acquisition of the fixed-ratio contingency and timeout period, as previously reported in rodent self-administration studies [24, 28–31]. There was also a main effect of days on infusions (*p* = 0.0119), and interaction between group and days (*p* = 0.0119), with no main effect of group on infusions, indicating group influenced intake patterns across days rather than infusions alone. As expected, yoked-cocaine animals did not exhibit consistent active nose-poke responding across sessions (SF1), reflecting the lack of behavioral control over drug delivery.

During extinction, there was a main effect of nose-poke (*p* = 0.0025, Fig. 1C, D), with no main effects for days, group, or interaction. Further validation showed a main effect of days between day 10, 11, and 20 (*p* = 0.0167, Fig. 1D), nose-poke (*p* < 0.0001), and days x nose-poke (*p* = 0.0006). Multiple comparisons showed differences between days 10 (*p* < 0.0001) and 11 (*p* = 0.0111) for nose-poke behavior, but this difference was no longer present by day 20, consistent with extinction learning. Separating active and inactive responses across days showed no difference between days 10 and 11 for active nose-pokes, but a reduction on day 20 relative to both day 10 (*p* = 0.0052) and day 11 (*p* = 0.0006), further supporting extinguished behavior. In contrast, inactive nose-pokes increased from day 10 to 11 (*p* = 0.0075) but showed no difference between days 10 and 11 compared to day 20.

Following extinction, we tested cue-induced reinstatement of cocaine seeking. During reinstatement, there was a significant effect of session on nose-poking behavior (*p* < 0.0001, Fig. 1E), Multiple comparisons showed significant increases in reinstatement 1 (*p* = 0.0008), reinstatement 2 (30 min *p* = 0.0191, 120-min *p* = 0.0031) with a significant decrease in nose-pokes of reinstatement saline (*p* < 0.0001) and no difference in behavior in yoked-cocaine animals. Consistent with the statistical plan (see methods), no sex differences were detected across behavioral, structural, and functional assays; data are therefore presented collapsed across sex (SF2, SF4, SF5).

### TdTomato and GFP tagging yielded identical spine morphometric results

**Fig. 2:**
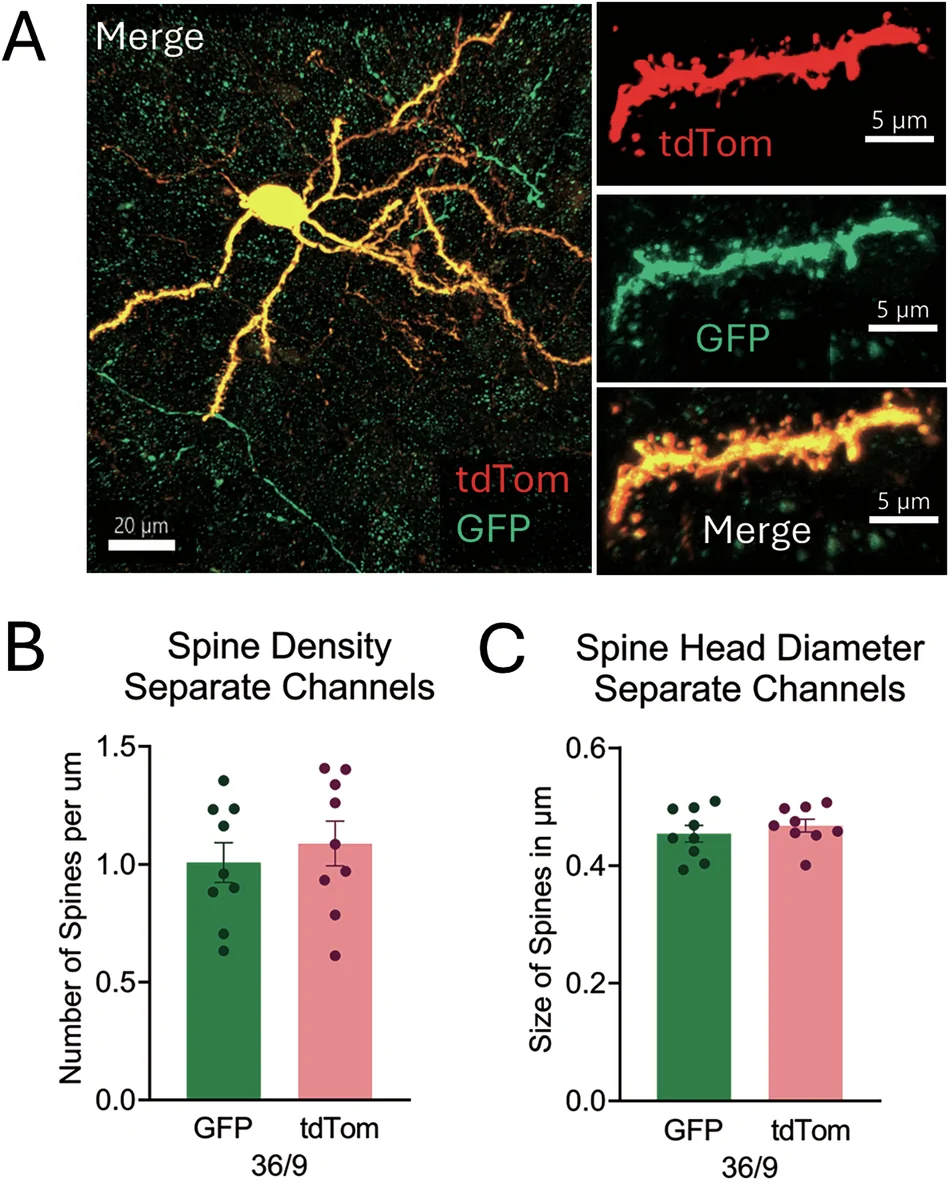
Validation of fluorescence tags being comparable. **A** Representative image of a neuron that expresses tdTomato (tdTom, red) and GFP (green) (left). Split of channels (right) showing spines in seperate channels, tdTom (top), and GFP (middle), merged image of a dendritic segment showing both channels (bottom). **B** Spine density on overlapping dendritic segments shows no difference based on channel. **C** Spine head diameter on overlapping dendritic segments shows no difference based on channel. Numbers below represent the total number of dendritic segments pooled across all animals in the group.

We next validated the anatomical labeling strategy used for structural analysis to compare ensemble and non-ensemble neurons. To validate that both tags are comparable, we first validated viral placement (SF3), then identified and assessed double-labeled neurons in cocaine self-administration animals (pooled at 0 and 30 min) for morphometric measurements in both channels. Confocal imaging confirmed that both tags filled the cell equally (Fig. 2A). Comparison between the two channels showed no differences in spine density (*p* = 0.5329; Fig. 2B) or spine head diameter (*p* = 0.4325).

### Neurons showed structural synaptic plasticity following 30 min of secondary reinstatement, which is enhanced in ensemble neurons

**Fig. 3:**
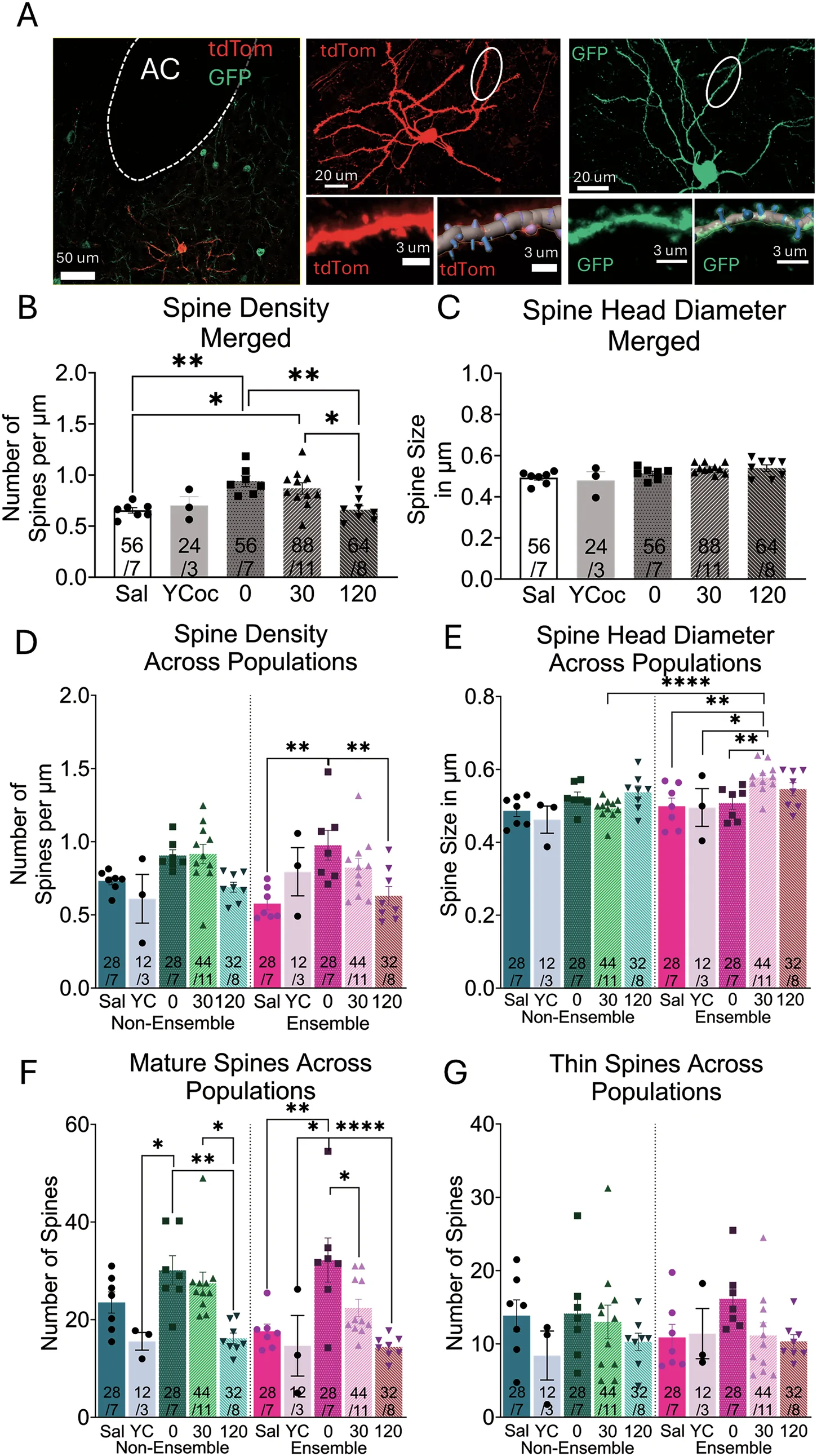
Structural characteristics of non-ensemble and ensemble neurons. **A** Left: representative images of nucleus accumbens core with non-ensemble GFP (green) and ensemble tdTomato (tdTom, red) neurons. Center: tdTomato neuron with measured segment indicated (top), with segment cropped (left) and segment after analysis (right). Right: GFP neuron with measured segment indicated (top), GFP segment cropped (left), and after analysis (right). AC anterior commissure. **B** All ensemble and non-ensemble dendritic segments together (merged) display changes in spine density. **p < 0.01 Yoked-saline (Sal), yoked-cocaine (YCoc), and extinguished (0-min) segments, *p < 0.05 yoked-saline and 30-min reinstatement segments. *p < 0.05 30-min and 120-min reinstatement segments. **p < 0.01 extinguished and 120-min reinstatement segments. **C** Merged dendritic segments exhibit no changes in spine head diameter. **D** Spine density across behavioral groups, Ensemble extinguished (0-min) compared to yoked-saline, and 120-min reinstatement segments, **p < 0.01. **E** Spine head diameter across behavioral groups. 30-min in the ensemble compared to ensemble yoked-saline and ensemble extinguished (0-min), **p < 0.01. Yoked-cocaine ensemble and 30-min ensemble reinstatement segments, *p < 0.05. Non-ensemble 30-min reinstatement compared to ensemble 30-min reinstatement segments, ****p < 0.0001. **F** Mature spines across behavioral groups. Non-ensemble yoked-cocaine compared to non-ensemble extinguished, *p < 0.05. Non-ensemble extinguished compared to 120-min reinstatement, **p < 0.01. Non-ensemble 30-min compared to 120-min reinstatement, *p < 0.05. Ensemble extinguished compared to ensemble yoked-saline, **p < 0.01. Ensemble extinguished compared to 120-min reinstatement, ****p < 0.0001, Ensemble extinguished compared to ensemble yoked-cocaine (YC), and 30-min, *p < 0.05. **G** Thin spines across behavioral groups. Numbers below represent the total number of dendritic segments pooled across all animals in the group.

We next examined the structural plasticity of ensemble and non-ensemble neurons by measuring spine head diameter and spine density at different key timepoints. For quantification, we analyzed single-tagged segments (Fig. 3A); both tagging types were pooled to test whether we could replicate previous results measuring all cells in the NAcore [16]. Spine density differed across groups (*p* = 0.0006; Fig. 3B), revealing increases from saline vs. extinguished (*p* = 0.0049), and from 30 min vs. 120 min (*p* = 0.0263). 120 min was different vs. extinguished (*p* = 0.0043) and 30 min (*p* = 0.0236). Yoked-cocaine animals did not have any differences in spine density from any other groups. Spine head diameter did not differ across groups (Fig. 3C).

We next compared spine morphology between ensemble and neighboring non-ensemble neurons. For spine density, there was no main effect of population (ensemble vs non-ensemble, Fig. 3D), but a main effect of group (yoked-cocaine, yoked-saline, extinguished 0-min, 30-min, and 120-min, *p* = 0.0006), and no interaction. Ensemble extinguished neurons had greater spine density than ensemble-saline (*p* = 0.0014), and 120-min (*p* < 0.0001), with no difference from ensemble yoked-cocaine. In contrast, no differences were observed across all non-ensemble groups. For spine head diameter, there were effects of population (*p* = 0.0182; Fig. 3E), group (*p* = 0.0227), and population × group (*p* = 0.0046). Specifically, ensemble 30-min spine head diameter was larger than ensemble saline (*p* = 0.0040), yoked-cocaine (*p* = 0.0429), extinguished (*p* = 0.0075), and 30-min non-ensemble counterparts (*p* < 0.0001). This change was not seen in non-ensemble neurons, indicating that ensemble neurons enlarged within 30 min of cue-induced reinstatement and did not return to baseline by 120 min.

### Spine classification revealed similar trends to mature spine destabilization across ensemble and non-ensemble neurons

To further characterize these structural changes, spines were classified as mature or thin (see Supplemental materials). In mature spines, there was a main effect of group (yoked-cocaine, yoked-saline, extinguished 0-min, 30-min, and 120-min, *p* < 0.0001, Fig. 3F), but no impact of population or interaction. Within non-ensemble neurons, a reduction in mature spines was observed comparing extinguished with 120 min (*p* = 0.0020), and yoked-cocaine (*p* = 0.0253), as well as from 30 min to 120 min (*p* = 0.0191). Ensemble neurons showed an increase in mature spines at extinguished from saline (*p* < 0.0001) and yoked-cocaine (*p* = 0.0047), and decreases from extinguished at 30 min (*p* = 0.0371) and 120 min (*p* < 0.0001), consistent with dynamic remodeling. Thin spines did not differ across groups (Fig. 3G). Spine head diameter did not correlate with seeking at 30 or 120 min (SF5A/B), suggesting that structural enlargement alone does not predict the behavioral magnitude of reinstatement behavior.

### Functional glutamatergic plasticity within ensembles and non-ensembles

**Fig. 4:**
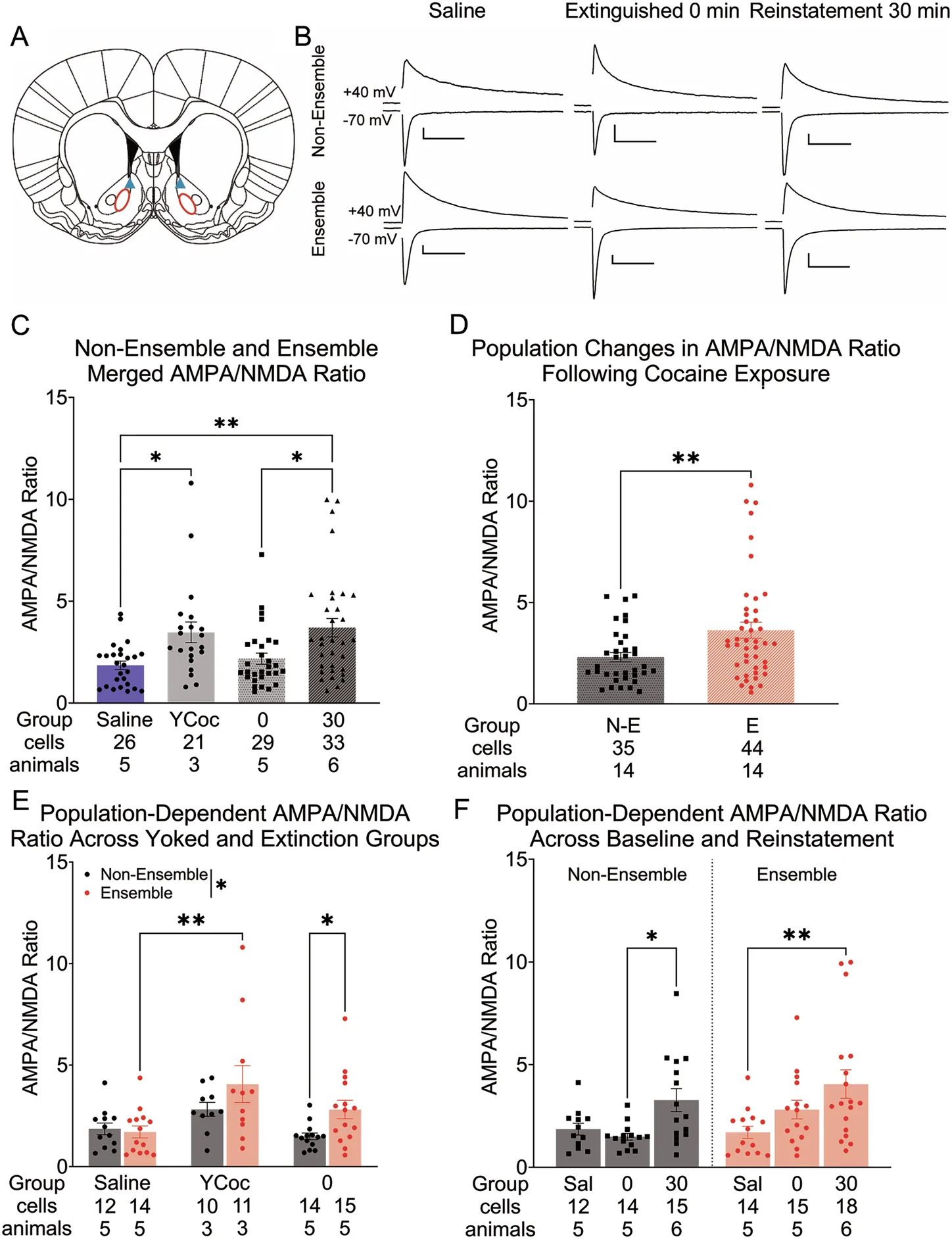
Functional plasticity alters following cocaine exposure. **A** Schematic of stimulating electrode (blue triangle) and patching area (red circle) for the nucleus accumbens core. **B** Representative traces of all behavioral groups, AMPA (top) and NMDA (Bottom). Minutes (min), scale bars: 100 pA/20 ms. **C** AMPA/NMDA ratios were significantly elevated from yoked-saline to yoked-cocaine, *p < 0.05, cocaine extinguished to reinstatement 30-min, *p < 0.05, and yoked-saline control animals to reinstatement 30-min, **p < 0.01. **D** Ensemble neurons (E) have an elevated AMPA/NMDA ratio compared to non-ensemble neurons (N-E) after cocaine exposure, **p < 0.01. **E** Cue-learning elevates AMPA/NMDA ratios in ensemble neurons greater than in non-ensemble neurons. Yoked-saline ensemble compared to yoked-cocaine ensemble, **p < 0.01, extinguished (0-min) ensemble vs non-ensemble, *p < 0.05. **F** Reinstatement elevates the AMPA/NMDA ratio based on population and group. Non-ensemble extinguished (0-min) compared to non-ensemble 30-min reinstatement, *p < 0.05, ensemble yoked-saline compared to ensemble 30-min reinstatement, **p < 0.01.

We next asked whether the structural changes observed in ensemble neurons were accompanied by functional alterations in excitatory synaptic transmission. We first combined ensemble and non-ensemble recordings to replicate previous reported increases in AMPA/NMDA ratio in the NAcore (Fig. 4A, B). Across populations, the AMPA/NMDA ratio differed across groups (yoked-cocaine, yoked-saline, extinguished 0 min, and 30 min) (*p* = 0.0009; Fig. 4C). Specifically, at 30 min, the AMPA/NMDA ratio was larger than saline (*p* = 0.0033) and extinguished (*p* = 0.0170). Yoked-cocaine cells had a larger AMPA/NMDA ratio than yoked-saline (*p* = 0.0327), but there were no differences between cocaine-exposed groups. Thus, rapid increases in the AMPA/NMDA ratio were observed after contingent and yoked-cocaine exposure.

We next compared cocaine-exposed (contingent and yoked) ensemble and non-ensemble neurons. When neurons were pooled across groups, ensemble neurons had a greater AMPA/NMDA ratio than non-ensemble neurons (*p* = 0.0081, Fig. 4D). We followed this by comparing the two yoked groups with extinguished animals that underwent self-administration to assess the effects of cue training and cocaine pharmacology on the AMPA/NMDA ratio (Fig. 4E). This comparison showed a main effect of group (*p* = 0.0019) and population (ensemble vs non-ensemble, *p* = 0.0321), with multiple comparisons revealing differences between the extinguished population of neurons (*p* = 0.0281) and differences between the ensemble yoked-cocaine and the ensemble yoked-saline (*p* = 0.0013). This further supports that cocaine exposure alone increases the AMPA/NMDA ratio selectively in the ensemble.

We next compared the baseline AMPA/NMDA ratio to the self-administration groups by neuronal population (Fig. 4F). When separated by group and population, a main effect of group (*p* = 0.0006; Fig. 4F) was observed, with no effect of population or interaction. In non-ensemble neurons, there was an increase in AMPA/NMDA ratio between extinguished and 30-min reinstatement groups (*p* = 0.0330). In contrast, ensemble neurons showed an increase from saline to 30 min (*p* = 0.0061), but not from extinguished conditions. No correlations were found with behavior (SF4D). Assessing presynaptic components using PPR revealed a group effect on PPR (*p* = 0.003), with no population or interaction effects (SF5). Multiple comparisons showed that yoked-cocaine animals had greater PPR than yoked-saline (*p* = 0.0031), extinguished (*p* = 0.008), and reinstated animals (*p* = 0.008). These findings suggest that presynaptic vesicle release probability was changed only by yoked-cocaine exposure and not operant cocaine intake.

Together, these findings show that AMPA/NMDA ratios vary across groups and are already elevated in cocaine exposed groups for ensemble neurons compared to non-ensemble neurons.

## Discussion

Cue-induced reinstatement induced distinct structural and functional adaptations across ensemble and non-ensemble neurons in the NAcore. Ensemble neurons exhibited increased spine head diameter during reinstatement, whereas non-ensemble neurons showed increases in AMPA/NMDA ratio. Because transient synaptic plasticity requires both structural enlargement and increased AMPA/NMDA ratio that returns to baseline, these findings indicate that ensemble neurons do not show transient synaptic plasticity during cocaine seeking. Plasticity changes were not accompanied by changes in PPR, regardless of whether neurons were classified as ensemble or non-ensemble, suggesting that presynaptic mechanisms are unlikely to contribute.

### Dissociable structural and functional synaptic adaptations in ensemble and non-ensemble neurons

Transient synaptic plasticity is well documented in non-selective NAcore neuronal populations during reinstatement. Previous studies reported rapid increases in both AMPA/NMDA ratio and dendritic spine head diameter that resolve within hours following cue exposure [13–15, 24, 29]. The present findings refine this framework by suggesting that structural and functional components of synaptic plasticity are distributed across distinct neuronal populations. We observed that ensemble neurons exhibit structural enlargement of dendritic spines, whereas non-ensemble neurons display an increase in the AMPA/NMDA ratio during reinstatement. Ensemble neurons may follow a distinct temporal trajectory of functional potentiation than non‑ensemble neurons, with structural changes emerging rapidly and functional changes occurring outside the 30‑min window examined here.

Expanding on structural findings, the change in spine head diameter was evident in ensemble neurons at 30 min of reinstatement from yoked-saline, yoked-cocaine, and extinguished ensemble neurons. Within the same animals, the spine head diameter increased at 30 min in ensemble neurons compared to non-ensemble neurons. This indicates that, at 30 min, ensemble neurons show spine head diameter changes previously associated with non-ensemble cells. Furthermore, spine classification analysis showed a reduction in mature spines following reinstatement, without a corresponding increase in thin spines. This pattern suggests cocaine seeking induces rapid remodeling of existing synapses [32]. Large mushroom and stubby spines, termed “mature” in this manuscript, are considered stable synaptic structures with strong synaptic connectivity and long-term memory storage [33, 34], and their modification may reflect destabilization or restructuring of established synaptic contacts during relapse-related circuit activation [35, 36]. These changes occurred in both ensemble and non-ensemble neurons, suggesting that while ensemble neurons display selective enlargement of spine head diameter, aspects of spine remodeling during reinstatement may occur more broadly across the NAcore neuronal populations.

Previous studies in male rats consistently reported increases in spine head diameter across non-selective populations during cued-seeking and drug-primed seeking [13–15, 24]. We did not detect such transient changes when pooling ensemble and non-ensemble neurons, likely due to methodological differences, including labeling strategy, species, and inclusion of females.

Functionally, we showed that ensemble neurons exhibited elevated AMPA/NMDA ratios following cocaine exposure even in the absence of reinstatement, suggesting that these neurons are already potentiated relative to non-ensemble neurons (Fig. 4D). This difference in AMPA-mediated signaling showed no transient increase post-cue-induced reinstatement compared to 0-min extinguished animals for ensemble neurons, but an increase compared to drug-naïve ensemble cells. Non-ensemble neurons increase in AMPA/NMDA ratio following 30 min of cue-induced reinstatement, replicating previous literature showing increased AMPA/NMDA ratio in cocaine rats [14, 24]. Importantly, assessing the AMPA/NMDA ratio with yoked-cocaine animals confirmed that cocaine exposure alone increases the ratio in a yoked setting, consistent with previous work utilizing non-contingent cocaine injections [37, 38]. When assessing PPR across groups, we observed a decrease in release probability following yoked-cocaine intake but not following operant cocaine intake. With the release probability remaining the same for yoked-saline and cocaine self-administration groups, it suggests that postsynaptic mechanisms, rather than changes in presynaptic release probability, underlie the observed plasticity.

Interestingly, structural spine enlargement was observed only in ensemble neurons, whereas functional measures of synaptic strength were not observed in ensemble neurons. One possible explanation is that AMPA/NMDA ratios primarily reflect changes in postsynaptic receptor composition and synaptic efficacy at existing synapses, whereas dendritic spine morphology provides a structural measure of synaptic remodeling and synapse formation [39]. Cocaine exposure is known to alter AMPA receptor trafficking and synaptic incorporation in the nucleus accumbens, thereby modifying synaptic strength without necessarily changing synapse number [40, 41]. Thus, ensemble neurons may undergo rapid structural remodeling during reinstatement, while functional changes in receptor composition may occur more broadly across NAcore neurons engaged during cue exposure.

Together, these findings suggest that structural potentiation in ensemble neurons does not produce detectable changes in the AMPA/NMDA ratio at 30 min. This may reflect (1) absolute changes in AMPA and NMDA receptor number that preserve their ratio, (2) input‑specific plasticity not captured by non-specific stimulation, or (3) ensemble‑specific structural remodeling that precedes functional potentiation within the timeframe studied. Thus, ensemble neurons appear to undergo structural remodeling during reinstatement without exhibiting the functional receptor changes that characterize transient synaptic plasticity.

### Study limitations and sex differences

While the present findings indicate that ensemble neurons undergo reinstatement-related structural plasticity, we also observed that non-ensemble neurons exhibit functional changes consistent with a background level of plasticity [16]. These findings support a model in which synaptic adaptations during reinstatement are not uniform across NAcore neurons but are instead compartmentalized within a functionally defined subset of neurons reactivated by drug-associated cues. In contrast to what might be expected if transient synaptic plasticity scaled with behavior, reinstatement intensity showed no significant correlation with synaptic plasticity metrics. This lack of association may reflect that ensemble plasticity encodes cue-associated network activity rather than the magnitude of behavioral output. Together with existing literature, the present structural data support the possibility that ensembles retain the capacity for rapid, cue-triggered spine remodeling. While the present study did not investigate whether ensemble neurons express D1 or D2 dopamine receptors, previous work using c-Fos-TRAP in self-administration showed that 66.7% of cocaine-seeking ensemble cells were D1-expressing medium spiny neurons [5]. Future work will be required to determine whether these ensemble-specific structural adaptations occur within defined cell-types and circuits to the NAcore, such as projections from the basolateral amygdala or prelimbic cortex that are known to drive cue-induced cocaine seeking [42, 43]. We also examined sex as a biological variable and observed no significant differences in structural or functional measures across male and female mice. However, the limited sample size of females due to breeding constraints warrants caution in interpretation. Given the well-documented sex differences in drug-seeking behavior and relapse susceptibility [44, 45], additional work is needed to detect potential sex-specific effects in ensemble plasticity.

## Conclusions

These findings suggest that reinstatement engages rapid structural plasticity within cocaine-seeking ensemble neurons. Functional synaptic changes occur in ensemble neurons with inherent potentiation after cocaine exposure, whereas non-ensemble neurons exhibit increased functional potentiation exclusively during cue-induced reinstatement. Together, reinstatement-associated structural plasticity occurs within mice cocaine-seeking neuronal ensembles in the NAcore.

## Supplementary information

Supplementary Materials, Methods, and Figures

Supplementary Table ST1

## Data Availability

The raw and analyzed data that support the findings of this study are available from the corresponding author upon reasonable request.
