# Supplementary material for: Synaptic plasticity in cocaine-seeking ensembles of the nucleus accumbens core
Source: Neuropsychopharmacology. 2026 May 2;51(11):2197–205. doi: 10.1038/s41386-026-02425-y (PMC13597511; doi:10.1038/s41386-026-02425-y)
Supplement: Supplementary file 1 [file 41386_2026_2425_MOESM1_ESM.docx]

**Supplementary Materials and Methods**

**Stereotaxic microinjections and catheter implantation**

All mice underwent stereotaxic and jugular catheter implantation surgeries at 8-22 weeks of age, performed under isoflurane anesthesia (induction: 3-5% v/v, maintenance: 1-2% v/v). Catheters (Silastic tubing ID 0.31 mm, OD 0.64 mm, Plymouth, MI, USA) were inserted 10-12 mm into the jugular vein post-anterior-cervical incision and sutured in place, with the infusion port positioned on the superior cranium using dental cement (CurioDental, Pikesville, MA, USA). Before the installation of the infusion port, 5 µl of non-specific pAAV-hSyn-EGFP (Addgene, Cat. # 50465-AAV2; Lot # V55182, Watertown, MA, USA) virus aliquots were diluted from 8.6 x 10^12^ with 15 µl of saline (ICU Medical, San Clemente, CA, USA) for a final concentration of 2.15 x 10^12^ and microinjected 300 nl (AP: +1.5 mm (mice < 25 g) or 1.6 mm (mice > 25 g); ML: ±1.0 mm, DV: 4.4) in the bilateral nucleus accumbens core of c-Fos-TRAP2 mice (n = 36). The injector was then raised by 0.01 mm and left in place for 8 minutes to allow virus dispersal before being slowly removed from the animal. Mice were then given 4 days to recover before behavioral testing began. Recovery was determined by tracking weight gain following surgery, with daily flushes of heparin (1 mg/kg, Fresenius Kabi, Bad Homberg, Germany), intraperitoneal injections of NSAID for analgesia and inflammation (carprofen, 5 mg/kg, MWI, Boise, ID, USA), and application of topical triple antibiotics (Medique Products, Fort Myers, Fl, USA). Mice used in functional cohorts received only catheterization and no microinjection (n = 19).

**Cocaine self-administration, extinction, and reinstatement**

**Operant chambers**

Experiments were conducted in standard mouse operant conditioning chambers (Imetronic, Pessac, France) equipped with two nose‑poke ports positioned on the front wall, each with an associated cue light. Chambers included a house light, an infusion pump, and a stainless-steel grid floor. The active port was paired with cocaine delivery and cue lights, while the inactive port had no programmed consequences. Yoked animals were tested in identical chambers to ensure comparable environmental conditions.

**Self-administration**

Mice self-administered intravenous cocaine (0.5 mg/kg/infusion, NIDA, Bethesda, MD, USA) or received yoked saline or cocaine in 2-hour daily sessions for 10 consecutive days in modular operant chambers equipped with two nose-poke ports and cue lights. The active nose-poke was paired with an availability cue light that remained illuminated until nose-poke activation, then extinguished for 10 seconds, and an internal nose-poke light that illuminated for 3 seconds immediately following nose-poke activation. The inactive nose-poke had no associated cues or programmed outcomes and served as a discrimination control. Yoked saline animals were paired to a cocaine self-administering “master” cage; they received a saline infusion whenever the master animal earned a cocaine infusion. Nose pokes by yoked animals did not produce infusions or alter cue lights. Mice were maintained on food restriction (80% of regular intake) during acquisition to facilitate operant responding [1–3].

**Extinction**

Following the acquisition, mice underwent a minimum of 10 extinction sessions (2 hours/day) with no cocaine delivery and no cocaine-paired cues. The extinction criterion was defined a priori as a decrease of at least 70% in active nose-poke responding relative to the mean of the last three days of self-administration, measured during the first 30 minutes of the extinction session. The first 30-minute window was selected because cue-induced seeking and early-session responding are most robust and provide a consistent time frame for cross-session comparison to reinstatement. To reduce injection-related responses, mice received sham intraperitoneal saline injections after each extinction session during the final three days of extinction.

**Reinstatement and TRAP2 tagging**

Cue-induced reinstatement sessions lasted 30 minutes and reintroduced the cocaine-paired cues without cocaine delivery. During reinstatement, the availability light was illuminated until active nose-poke responses, then extinguished for 10 seconds, while the internal nose-poke light illuminated for 3 seconds after each active nose-poke. Immediately after reinstatement, mice received intraperitoneal 4-hydroxy-tamoxifen (4‑OHT; 50 mg/kg) to activate Cre-dependent TRAP2 [5] labeling of activity-defined ensemble neurons with tdTomato. Ensemble neurons were defined as tdTomato-positive (tdTomato+) cells labeled during reinstatement 1. A minimum of 4 additional extinction sessions followed the initial reinstatement to allow tdTomato expression to extend into dendrites and spines. Cohorts were then sacrificed immediately after a second reinstatement at 30 or 120 minutes. Extinguished animals, as well as yoked-saline and yoked cocaine-control animals, were sacrificed from their home cage without additional chamber exposure, but were otherwise handled identically to reinstatement 2. To confirm that c-Fos-TRAP2 labeling required 4‑OHT, a separate cohort of mice underwent conditioned place preference testing without 4‑OHT administration and showed no tdTomato expression (data not shown).

**Immunohistochemistry**

Mice in the structural cohorts from p88-186 were anesthetized with a 2:1 combination of ketamine (MWI) and xylazine (Akorn, Lake Forest, IL, USA) and then perfused with 10 mL of 1X phosphate-buffered saline (PBS, Quality Biological, Gaithersburg, MD, USA) and 20 mL of 3.7% formaldehyde (Fisher Scientific, Waltham, MA, USA). Brains were post-fixed with 3.7% formaldehyde for at least 24 hours and then transferred to a solution of 20% sucrose, 0.01% sodium azide (Sigma-Aldrich, Burlington, MA, USA) in 1X PBS. Brains were then sectioned using a cryostat (Leica, Wetzlar, Germany) at 80 μm to maximize the ability to keep distal dendrites intact. Free-floating sections were then rinsed 3 times in 1X PBS and incubated for 2 hours in a blocking solution of 5% normal goat serum (NGS, Invitrogen Waltham, MA, USA), 2.5% bovine serum albumin (BSA, Fisher Scientific), and 0.25% Triton x 100 (Thermo Fisher Scientific, Waltham, MA, USA) in PBS, then incubated in this blocking solution plus the primary antibody for 12-16 hours to enhance fluorescent signal. Rabbit anti-RFP primary antibody (Rockland Immunochemical, 1:1000 dilution) and chicken anti-GFP primary antibody (Aveslabs, 1:10,000 dilution, Davis, CA, USA) were used. The tissue was then rinsed once in 0.25% PBS-Triton, three times in 1X PBS, and incubated for 2 hours in secondary antibodies: Goat anti-rabbit IgG, Alexa Fluor 594 (Thermo Fisher Scientific, 1:1000 dilution), and donkey anti-chicken IgY, Alexa Fluor 488 (Thermo Fisher Scientific, 1:1000 dilution). The tissue was then mounted onto slides using Prolong Gold mounting medium (Thermo Fisher Scientific).

**Acquisition and quantification of dendritic spines**

High-resolution images of 80 µm NAcore slices were acquired using a 63x 1.4 NA, oil immersion objective on a Zeiss LSM 880 (Colorado State University) or 980 (University of Wyoming) confocal microscopes (ZEISS Oberkochen, Germany) with voxel size set to 0.164 µm at the XY plane and 0.25 µm intervals along the z-axis with a frame set to 1024x1024 pixels. Medium spiny neurons within the NAcore region, located between Bregma 1.7 mm and 0.62 mm (Paxinos, 2007, 3rd ed.), were identified in Alexa 594 and Alexa 488 laser channels and excited with a 488 nm laser for GFP and a 561 nm laser for tdTomato. Images were converted into Imaris file format using the Imaris file converter. To reduce sample bias, four segments per animal were included for each neuron classification. Using researchers blinded to treatment conditions, dendritic segment images were cropped in Imaris (versions 10.0.0, 10.0.1, Oxford Instruments, Zurich, Switzerland) starting at least 50 µm from the soma after the first branch point, and segment lengths were 30 - 110 µm for analysis. Following the cropping of the dendritic segments, the images were then cleaned of background using the "surfaces" function in Imaris. Dendritic spines were then identified and quantified using the built-in Imaris filament tracing algorithm for filaments programmed with the following parameters: starting point, largest diameter = 1.30 µm, dendrite seed point thinnest diameter = 0.395 µm, diameter of sphere region = 1.30 µm, spine seed point thinnest diameter = 0.217 µm (max length 6.58 µm).

**Spine classification**

Following the creation of the analyzed segments, we then followed up by utilizing the Imaris XT plugin, Classify Spines [6]. We used the following parameters for spine classification, stubby spines: spine length less than 1 um, Mushroom: length of spine less than 3 um and max spine head width greater than mean spine neck width *2, thin spine mean spine head greater than or equal to mean spine neck width, and filipodia/dendrite is all other spines that did not fall into the above category. Note that we only used the classification for stubby, mushroom, and thin spines, but still utilized the filipodia/dendrite-labeled spines for spine density and d_h_ metrics, as they did not fit into any of the previous criteria but were identified as spines by the experimenters.

**Whole cell recordings**

Acute brain slices of the NAc for electrophysiology were prepared from p85 to p117 male and female mice. Mice were anesthetized using isoflurane (2-3%) until unresponsive to toe-pinch and then transcardially perfused with ~10 mL of cold (4°C), oxygenated cutting solution which contained (in mM): 23 NaCl, 3.5 KCl, 1.25 NaHPO_4_, 26 NaHCO_3_, 1 MgCl_2_, 1.5 CaCl_2_, 10 D-glucose (290–310 mosmol, pH 7.3). Coronal slices (250-300 μm), which included the nucleus accumbens, were prepared using Compresstome (VF-510-0Z, Precisionary Instruments, Ashland, MA, USA). Slices were allowed to recover for at least 30 minutes in warm (37°C) aCSF, which contained (in mM): 123 NaCl, 3.5 KCl, 1.25 NaHPO_4_, 26 NaHCO_3_, 1 MgCl_2_, 1.5 CaCl_2_, 10 D-glucose (290–310 mosmol, pH 7.3).

Whole-cell voltage clamp recordings from both unlabeled and tdTom cells in the NAcore were made using an Olympus BX51WI microscope (Tokyo, Japan) using CoolLED (550 nm) optical stimulation to identify tdTom cells. To isolate excitatory synaptic transmission, the extracellular solution included 20 μM SR95531 (Tocris Biosciences, Bristol, United Kingdom) to block GABAA receptors. Voltage clamp recordings were made using a Cs-gluconate internal solution, which contained (in mM): 120 CsCH_3_SO_3_, 3 NaCl, 2 MgCl_2_, 1 EGTA, 10 HEPES, 4 MgATP, 0.3 Tris-GTP, 14 Tris-creatine phosphate, 12 sucrose (288 mosmol, buffered with CsOH to pH 7.32). Borosilicate patch pipettes were pulled to 2–5 MΩ using a Narishige vertical puller (Tokyo, Japan). The liquid junction potential (10 mV) was corrected during recording. Temperature was maintained at 35–37°C by a Warner TC-324B controller. Data were digitized at 20 kHz and filtered at 10 kHz. Data was acquired with a Sutter Instruments IPA amplifier/digitizer using the SutterPatch software (IgorPro, Wavemetrics, Lake Oswego, Oregon). Before and after data acquisition, access resistance was recorded using a 10-mV hyperpolarizing step, and recordings with changes > 20% across the recording were discarded.

To stimulate cortical inputs to the nucleus accumbens, a bipolar stimulating electrode was placed near the NAcore, generally medial/dorsal to the anterior commissure. To stimulate synaptic transmission, a constant current (0.1 ms, 2-32 mA) was applied to the slice. Synaptic responses were recorded at -70 mV and +40 mV to record both AMPA and NMDA receptor contributions. EPSCs were generated at an inter-stimulus interval of 10 seconds, and ≥10 sweeps at each holding potential were averaged together. Data were analyzed using SutterPatch (Sutter Instrument, Novato, CA, USA) and IgorPro software. EPSCs at -70 mV and +40 mV were averaged and baseline-subtracted. To estimate the A/N ratio, we measured the peak EPSC amplitude at -70 mV and divided it by the amplitude at +40 mV measured at 40 ms after stimulus onset, to isolate the NMDA receptor component.

**Summary of excluded animals and cells**

Exclusion criteria included catheter failure (2 animals), lack of tdTomato expression (2 animals), failed discrimination (2 animals), mislocalized cells (2 cells in recordings), and statistical outliers (ROUT, Q = 1%) (3 cells in non-ensemble recordings). Non‑responding cells were excluded from PPR but retained for A/N analysis.

**Supplemental Table 1. Full Statistical Results**

Provided as a separate Excel file per Neuropsychopharmacology guidelines.

**Supplemental Results**

**Sex comparisons: self-administration**

Separating the animals in Figure 1 by sex reveals a main effect of days (F (2.971, 101) = 8.062, p < 0.0001), nose pokes (F (1.000, 34.00) = 135.9, p < 0.0001) and days x nose pokes (F (2.635,89.60) = 6.678, p = 0.0007), with no main effect of sex and interaction with sex.

**Sex comparisons: extinction**

Assessing extinction by sex revealed a main effect of only nose pokes (F (1.000, 35.00) = 6.013, p = 0.0193) with no main effect of days, sex, or interactions.

**Sex comparisons: reinstatement**

Examining the reinstatement of animals by sex revealed a reinstatement between males initially at reinstatement 1 (p = 0.0002), reinstatement 2 at 30 minutes (p = 0.0002), with no significant reinstatement 2 at 120 minutes. Females showed a trending initial reinstatement (p = 0.0526), but did not exhibit reinstatement at any point.

**Supplementary Figures**

Supplemental Figure 1. c-Fos-TRAP2 mice during yoked-cocaine self-administration behavior. (**A**) Yoked-cocaine self-administration timeline. Animals were injected with 4-hydroxy-tamoxifen (4-OHT) following the first reinstatement. (**B**) Nose-poke comparisons reveal no differences in active and inactive nose pokes. (**C**) Extinction sessions showed no difference in nose-pokes.

Supplemental Figure 2. Male and female c-Fos-TRAP2 mice during cocaine self-administration behavior. (**A**) Cocaine self-administration timeline for males and females. Animals were injected with 4-hydroxy-tamoxifen (4-OHT) following the first reinstatement. (**B**) c-Fos-TRAP2 mice infusions, no difference between groups on infusion rate (**C**). Sex comparisons reveal no differences in active and inactive nose pokes. **** p < 0.0001 Active vs inactive nose-poke within sexes. (**D**) Extinction sessions showed no difference in sex on nose-pokes. (**E**) Active nose-pokes following cue-induced reinstatement for cocaine were elevated only within male mice with no difference between sex on the reinstatement level, **** p < 0.0001 male extinction active nose-poke compared to first reinstatement active nose-pokes, *** p < 0.001, male extinction active nose-pokes compared to second reinstatement 30 minutes, * p < 0.05, male extinction active nose-pokes compared to second reinstatement 120 minutes.

Supplemental Figure 3. Virus tract locations in the nucleus accumbens core. (**A**) Representative 20x image of GFP virus tract and injection site. Anterior commissure (AC) (**B**) Atlas images of virus injection sites. X denotes virus injection sites from 36 animals, modified from Paxinos and Franklin's mouse brain in stereotaxic coordinates, 5^th^ edition.

Supplemental Figure 4. Sex comparisons reveal no difference in spine morphology. (**A**) Spine density does not differ across sexes. Yoked-cocaine (YCoc), Extinguished (0 minute). (**B**) Spine head diameter does not vary across sexes. (**C**) The number of mature spines does not vary across sexes. (**D**) The number of thin spines does not vary across sexes.

Supplemental Figure 5. Spine head diameter and AMPA/ NMDA ratio do not correlate with seeking on reinstatement 2. (**A**) Correlation of 30-minute reinstatement 2 to spine head diameter. (**B**) Correlation of 120-minute reinstatement2 to spine head diameter. (**C**) sex comparisons of the AMPA/NMDA ratio. Yoked-cocaine (YCoc). (**D**) Correlation of 30-minute reinstatement 2 to ensemble and non-ensemble AMPA/NMDA ratio.

Supplemental Figure 6. Paired pulse ratio of ensemble and non-ensemble neurons at different timepoints. (**A**) Representative traces of paired pulse data from all cell types and time points. Minutes (min) scalebars 200 pA / 20ms. (**B**) Differences in paired-pulse ratio based on groups. *** p < 0.001, ** p < 0.01. Red dots denote ensemble neurons, black squares mark non-ensemble neurons. Yoked-cocaine (YCoc), extinguished (0 minute).

**References**

1.	Horenstein BR. Performance of conditioned responses as a function of strength of hunger drive. Journal of Comparative and Physiological Psychology. 1951;44:210–224.

2.	Leslie JC. Effects of Food Deprivation and Reinforcement Magnitude on Conditioned Suppression. Journal of the Experimental Analysis of Behavior. 1977;28:107–115.

3.	Henton WW, Fisher BR. Cyclical Food Deprivation and Classical Conditioned Response Patterns. Psychol Rec. 1981;31:377–393.

4.	Bobadilla A-C, Dereschewitz E, Vaccaro L, Heinsbroek JA, Scofield MD, Kalivas PW. Cocaine and sucrose rewards recruit different seeking ensembles in the nucleus accumbens core. Mol Psychiatry. 2020;25:3150–3163.

5.	DeNardo LA, Liu CD, Allen WE, Adams EL, Friedmann D, Fu L, et al. Temporal Evolution of Cortical Ensembles Promoting Remote Memory Retrieval. Nat Neurosci. 2019;22:460–469.

6.	Sebastian V, Estil JB, Chen D, Schrott LM, Serrano PA. Acute Physiological Stress Promotes Clustering of Synaptic Markers and Alters Spine Morphology in the Hippocampus. PLOS ONE. 2013;8:e79077.
